# Editorial: Carbohydrate-Based Molecules in Medicinal Chemistry

**DOI:** 10.3389/fchem.2021.655200

**Published:** 2021-03-05

**Authors:** Nuno M. Xavier, Peter R. Andreana, Ivone Carvalho, Mark von Itzstein

**Affiliations:** ^1^ Centro de Química Estrutural, Faculdade de Ciências, Universidade de Lisboa, Lisboa, Portugal; ^2^ Department of Chemistry and Biochemistry, School of Green Chemistry and Engineering, University of Toledo, Toledo, OH, United States; ^3^ School of Pharmaceutical Sciences of Ribeirão Preto, University of São Paulo, Ribeirão Preto, SP, Brazil; ^4^ Institute for Glycomics, Griffith University, Gold Coast, QLD, Australia

**Keywords:** carbohydrate chemistry, glycomimetics, glycoconjugates, heptoses, chitosan oligosaccharides, molecular recognition, micro/nanocapsules, drug delivery

The therapeutic potential of carbohydrates has been well demonstrated by the number of carbohydrate-based compounds, either synthetic, semi-synthetic or from natural sources for the treatment of disease states such as bacterial and viral infections, cancer or diabetes, to name only a few. The discovery of bioactive carbohydrates and their development toward a further pharmaceutical application involve interdisciplinarity studies bridging chemistry, biology, and pharmaceutical/medical sciences, such as carbohydrate chemistry, biochemistry, glycobiology, drug design, microbiology and oncology. The biological profile of these molecules arises by a variety of mechanisms, many of them relying on the interference with carbohydrate-dependent events, including the inhibition of carbohydrate-acting enzymes, inhibition of carbohydrate-protein or carbohydrate-carbohydrate interactions, interaction with biologically relevant carbohydrate-based structures or inhibition of their biosynthesis. Carbohydrate-based molecules offer additional important benefits which further enhances their pharmaceutical and biomedical interest, namely their polyfunctionalized nature and stereochemistry, which enable for a fine tuning and modulation of the biological and pharmacokinetic properties, their hydrophilicity, biocompatibility, and low toxicity. The articles included in this Research Topic illustrate how well carbohydrates may provide and contribute to ground-breaking therapeutic solutions, using novel carbohydrate chemistry strategies.

Stanetty and co-workers (Suster et al.) describe an elegant and efficient method for the differentiation of the substitution patterns of L-*glycero*- and D-*glycero*-D-*manno*-heptopyranose scaffolds. These units are present in the inner core-region of bacterial lipopolysaccharides (LPSs), which are crucial structures of the outer membrane of Gram-negative bacteria and essential for the recognition of bacteria by the host immune system. Therefore, targeting the components of LPSs is a strategy of utmost interest for the development of novel drugs and vaccines, aside from potentially offering new opportunities for circumventing antibiotic resistance. The authors present a simple and versatile methodology for accessing D,D- and L,D-heptosides with all positions differentially substituted, relying on exocyclic 1,2-diol protection of the fully hydroxylated heptoside using the tetraisopropyl disiloxyl (TIPDS) group and selective protection of the C-2 and C-3 hydroxyl groups as an orthoester. The strategy allows for the differentiation of sugar ring substituents by a sequence of *O*-acylation at C-4, orthoester hydrolysis, which occurs with selective C-2-esterification, and *O*-acylation of C-3. The exocyclic substituents are then differentiated by a regioselective cleavage of the 6,7-*O*-TIPDS moiety, leading to a 7-OH acceptor, enabling the complete differentiation of the heptoside scaffold and providing a template which can be useful for oligoheptoside formation.

The contribution of Nifantiev and co-workers (Tsvetkov et al.) reports the synthesis of a series of chitosan-based oligomers and evaluation of their immunomodulative activities, along with those related to chitin counterparts. Chitin, a polysaccharide of β-(1→4)-linked *N*-acetyl-D-glucosamine polysaccharide is an essential constituent of the inner cell wall of fungi. Both chitin and its fully or partially *N*-deacetylated form, chitosan, possess immunomodulatory effects, being recognized by the human immune system and eliciting various immune responses. The synthetic pathway includes the preparation of a disaccharide thioglycoside donor comprising a 4′-*O*-*tert*-butyldimethylsilyl (TBDMS) group, which is accomplished via orthogonal glycosylation of a 4-OH *N*-phthaloyl-protected glucosamine-derived thioglycoside with a 4-*O*-TBDMS *N*-phthaloyl-protected glucosamine trichloroacetimidate. Glycosidation with a 4-OH-related azidoethyl glycoside, and reiterative desilylation/glycosylation then allows for further oligosaccharide assembly. Three chitosan oligosaccharides containing 3, 5 and 7 glycosyl units were synthesized and converted into the corresponding biotinylated products. Biological evaluation revealed that the synthetic chitosan and chitin oligosaccharides promote an effective immune response and immunomodulation in RAW 264.7 cells with an increased proliferation of the macrophages, phagocytic activity and cytokine production; the latter being more notorious when cells were exposed to chitosan oligosaccharides. In addition the synthetic oligosaccharides did not show antiproliferative and cytotoxic effects, which indicates their suitability as epitopes for activation of the immune system.

Glycoconjugates are essencial for recognition and adhesion in mammal cells, which contribute to inflammation, cell abnormal proliferation and metastasis, and for host cell-pathogen recognition and adhesion; the latter events being essential for initiating infection. Moreover, abnormal glycosylation patterns are observed in cancer cells. These aspects turn the synthesis of synthetic glycoconjugates highly relevant in the context of drug discovery, glyco-vaccine development as well as for their use as biological probes. A review by Sarkar and Jayaraman compiles chemical methods for connecting carbohydrates to proteins, lipids and oligonucleotides. Glycoconjugation methodologies based on amidation, reductive amination, native chemical ligation, Staudinger ligation, azide-alkyne cycloaddition, thiol-ene conjugation, disulfide bond formation, Suzuki-Miyaura coupling, cross metathesis, Michael addition to glycosyl vinyl sulfones and to sugar vinyl sulfoxides are thoroughly discussed. In addition, the effect of the glycoconjugation on the properties of the biomolecules is covered.

A topic that has attracted much interest is the development of inhibitors/antagonists of carbohydrate-binding proteins (lectins). The contribution of Jiménez-Barbero and co-workers (Bertuzzi et al.) gives an overview on the development of inhibitors of galectins, lectins that bind to β-galactoside-containing structures, which are involved in various pathological conditions. The survey gives a special emphasis on human galectins 1 and 3 and covers different types of inhibitors, including approaches used to increase affinity, and encompassing monovalent and multivalent glycomimetics. Different types of structures such as *C*-glycosyl compounds, thiogalactosides or multimeric lactosides bearing key functional groups and moieties, such as aryl, triazole, or aryltriazole-units, and the influence of structural aspects in binding affinity and selectivity are also discussed.

The ability of carbohydrates to interact with cell-surface receptors aligned with their typical characteristics, *i.e.,* solubility and biodegradability, also make them attractive molecules for the construction of drug delivery systems. Bielski, Witczak et al. describe in their review, a new and robust method for interfacial polymerization leading to micro-and nanocapsules from carbohydrate-based building blocks. The novelty of the strategy, named by the authors as IPCESCO (*Interfacial Polymerization for Capsules’ External Surface Control*) allows for controlling the density of required chemical functionalities and moieties on the external capsule’s surface. The variability and scope of the method, including the possible monomers, such as modified mono- and disaccharides, and their synthesis, and the transformations of the external capsule’s surface functional groups into the desired moieties, are covered. Among the prospective biomedical applications of IPCESCO-constructed micro/nanocapsules, their use in drug delivery is highlighted. For this purpose, the selected capsule’s external moieties may enable overcoming several limitations, such as reducing the degradation of the capsule and the active pharmaceutical ingredients, preventing formation of aggregates, and facilitate the crossing of biological barriers.

We hope the articles published in this Research Topic will encourage researchers to embrace or to continue discovering and exploring the stimulating contributions that carbohydrates may bring to medicinal and pharmaceutical chemistry!

